# Galvanic vestibular stimulation for postural rehabilitation in neurological disorders: a systematic review

**DOI:** 10.3389/fnins.2025.1580078

**Published:** 2025-04-16

**Authors:** Wei Fu, Ya Bai, Xiaoming Wang

**Affiliations:** ^1^Department of Geriatrics, Xijing Hospital, Air Force Medical University, Xi’an, China; ^2^Department of Neurology, Xijing Hospital, Air Force Medical University, Xi’an, China

**Keywords:** galvanic vestibular stimulation, rehabilitation, vestibular, neurological disorder, neuromodulation

## Abstract

**Background:**

Galvanic vestibular stimulation (GVS) may potentially improve postural rehabilitation. However, the postural control role of GVS in the neurological disorders has not been systematically reviewed.

**Methods:**

We conducted a systematic review on PubMed, EMBASE, and Web of Science to synthesize key findings of the effectiveness of single and multiple sessions of GVS alone and combined with other interventions on balance in adults with neurological disorders. Diagnosis of neurological disorders, sample size, age and gender of participants, GVS parameters, postural assessments, and study findings were extracted following the PRISMA guidelines. Newcastle-Ottawa scale was used to assess study quality.

**Results:**

Twenty-five studies were included in the systematic review. Clinical application of GVS for postural control included Parkinson’s disease, bilateral vestibulopathy, stroke-induced hemiplegia, multiple sclerosis, progressive supranuclear palsy, persistent postural-perceptual dizziness, and unilateral vestibulopathy. GVS effectively improves postural control in most neurological disorders. Risk of bias assessment showed that most studies had a low risk of bias.

**Conclusion:**

GVS is a promising complementary therapy to improve postural control and balance in adults with neurological disorders. Future high quality studies should be performed to confirm these findings.

## Introduction

Galvanic vestibular stimulation (GVS) stands out as a burgeoning neuromodulation approach, offering a non-invasive means for activating this vestibular system effectively (Fitzpatrick et al., 1999). This primary reason behind choosing the vestibular system as a promising site for therapeutic stimulation in the management of diverse ailments lies in its extensive and intricate connectivity with numerous brain structures, as evidenced by the tortuous course of the vestibular pathway. This procedure stimulates semicircular canals, otolith organs, and contiguous vestibular nerves, ultimately connecting to this vestibular nuclei situated within a brainstem. These nuclei maintain a dialog with these thalamic relay stations, notably the ventral posterolateral nucleus. It is from this relay hub that ascending vestibular pathways would establish synaptic connections with various vestibular cortical regions, encompassing these central sulcus, somatosensory cortex, the parietal lobe, and the insular parietal vestibular cortex (Iles et al., 2007). Regarding these descending ways, stimulus is conveyed to these vestibulospinal, reticulospinal, and corticospinal tracts within spinal cord, thereby eliciting a postulate reaction (Liechti et al., 2008).

Building upon the advancements in stimulation electrode technology and the refined stimulus dynamics in recent years, bipolar GVS was employed as a non-invasive technique to generate these vestibular organs (Fitzpatrick and Day, 2004). Surface electrodes are securely affixed to the mastoid bones, and an electrical stimulus is administered, typically defined by a low-intensity pulsed direct current, with a cathode placed upon one mastoid process and an anode situated on the diametrically opposite side. It is acknowledged that perilymphatic cathodal currents have the effect of depolarizing the trigger site, which in turn facilitates excitation, whereas anodal currents induce hyperpolarization at the site, leading to inhibition (Gensberger et al., 2016). Electrical currents utilized in GVS are commonly administered in the form of incremental steps, sinusoidal waves, brief pulses, or constrained bandwidth noise (Dlugaiczyk et al., 2019). A comprehensive assessment of the distinct parameters that can be adjusted within the GVS waveform has revealed a wide spectrum of configurations has been employed (McLaren et al., 2023).

At present, GVS has garnered extensive research interest and clinical applications, owing to its attributes of safety, user-friendliness, affordability, rapid efficacy, and the minimal discomfort it causes to patients. Previous research has clearly shown that GVS treatment significantly enhances vestibulospinal function, thereby stabilizing the disrupted postural and balance control in patients suffering from vestibular disorders, such as bilateral vestibulopathy (Wuehr et al., 2023), UVP (Ceylan et al., 2021), and vestibular dysfunction of elderly adults (Fujimoto et al., 2016). Besides, GVS also improves postural instability, bending posture, trunk rigidity, and akinesia in neurological diseases, like Parkinson’s disease (PD) (Kataoka et al., 2022), stroke (Tomioka et al., 2022), and complete spinal cord injury (Čobeljić et al., 2018). However, there has yet to be a systematic review focusing on postural rehabilitation of GVS in neurological disorders. Therefore, the aim of this review is to undertake a systematic scrutiny of pertinent literature pertaining to the postural restoration of GVS in patients with neurological conditions.

## Methods

### Search strategy

**Figure 1:**
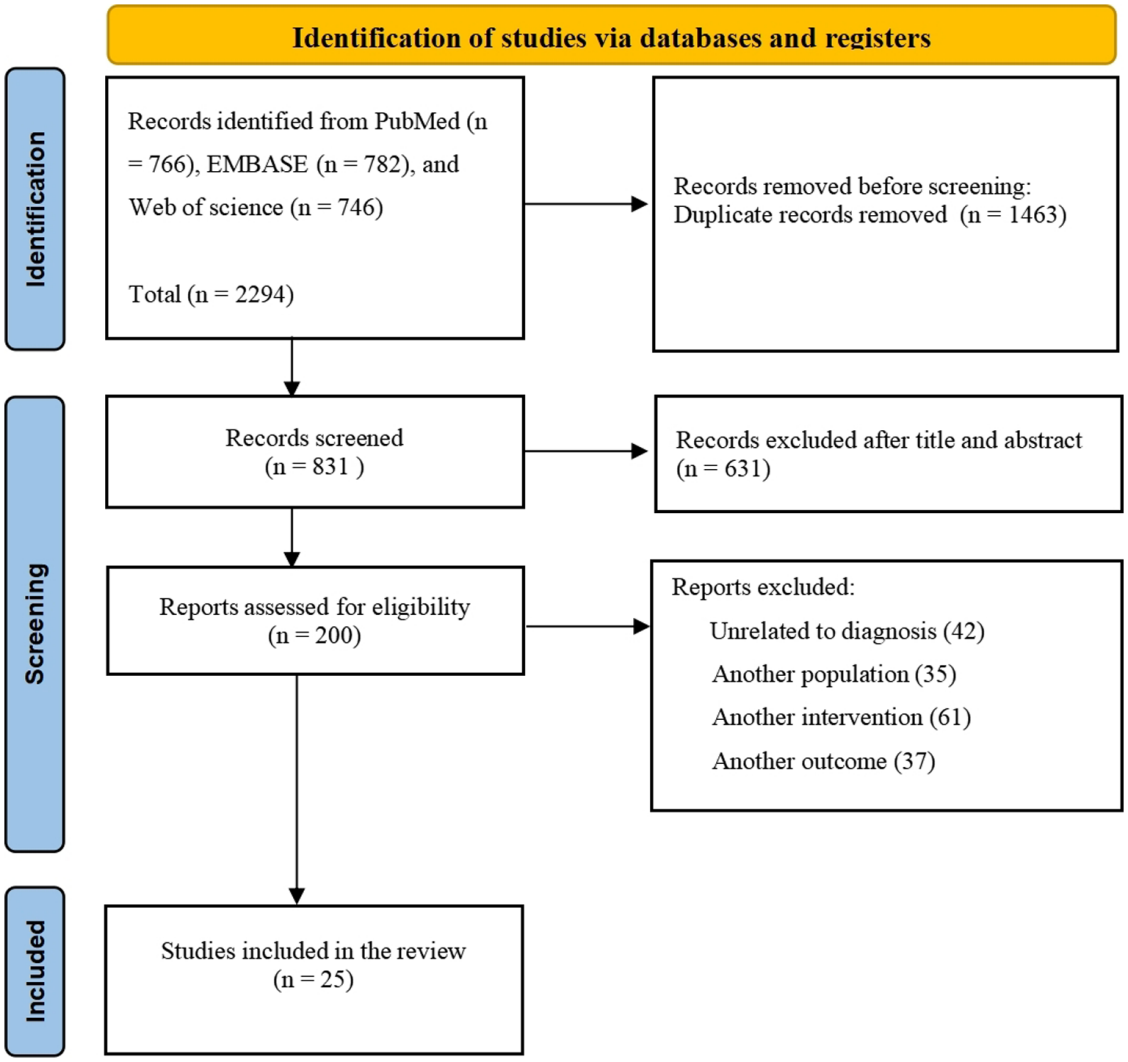
PRISMA flowchart showing the screening process.

We conducted a thorough search across PubMed, EMBASE, and Web of Science. Our search strategy encompassed a comprehensive approach: (“galvanic vestibular stimulation” OR “GVS” OR “vestibular electrical stimulation” OR “non-invasive brain stimulation”) AND (“postural control” OR “postural responses” OR “postural function” OR “postural stability” OR “static balance” OR “postural adjustments” OR “postural balance” OR “postural equilibrium” OR “postural sway”). English-language studies from database inception through 25 September 2024 were inclusive. This meticulous examination was meticulously conducted in accordance with the PRISMA criteria, as illustrated in Figure 1.

### Eligibility criteria

The outlined inclusion criteria were applied in the selection process of the original articles for thorough analysis: (1) adults with neurological disorders, (2) GVS alone or combined with another intervention, (3) incorporated at least one assessment of posture or equilibrium as a result measure, and (4) randomized and non-randomized controlled trials, observational studies, and experimental methodologies. The selection criteria involved excluding the following types of publications: reviews, those not in English, case reports, letters, editorial pieces, articles pertaining to children, as well as books.

### Data extraction and analysis

Data from individual studies were meticulously gathered by two independent authors. Initially, the title and abstract of the studies were screened for title, abstract and keywords, if the study was eligible, the full text was reviewed and read to check whether they met the inclusion criteria. We extracted information on authors, year of publication, diagnosis of neurological disorders, sample size, age and gender of participants, GVS parameters, postural assessments, and study findings. Disagreements were resolved by a third experienced author through a full-text review and double-blind data extraction.

### Methodological quality assessment

All manuscripts were meticulously assessed for their methodological rigor employing an adapted version of Newcastle–Ottawa Scale (NOS) (Stang, 2010). The assessment for these studies’ quality was conducted across five distinct dimensions: the representativeness of the sample, the rigor of intervention protocol, the homogeneity in comparability, the clarity of these outcome measures, and the robustness of statistical analysis. A score of 5 represents the pinnacle of quality, with ascending scores signifying an enhanced level of research excellence. Based on the cumulative points allocated, each research endeavor was evaluated as having a low risk of bias (≥3 points) or a high risk of bias (≤3 points). This methodological rigor of these studies was evaluated independently through two reviewers, and any discrepancies in their judgments were settled through collaborative discussion and mutual agreement.

## Results

### Study selection and included studies characteristics

Upon conducting an initial review, executing full-text searches, and meticulously verifying the references cited within the selected articles, a compilation of 25 studies was meticulously identified as conforming to the predefined inclusion criteria, thus meriting inclusion in the subsequent analysis. The intricacies of the search and screening protocol are delineated in Figure 1, which illustrates the PRISMA flowchart delineating the inclusion process.

### Description of included studies

Table 1 delineates the demographic composition and defining attributes of the 25 studies incorporated into this systematic review, which span from 2009 to 2024. Notably, seven of these studies were carried out involving participants with PD (Wuehr et al., 2022; Pal et al., 2009; Samoudi et al., 2015; Peto et al., 2024; Kataoka et al., 2016; Khoshnam et al., 2018; Okada et al., 2015), 12 with BVP (Wuehr et al., 2023; Ko et al., 2020; Eder et al., 2022; Schniepp et al., 2018; Iwasaki et al., 2018; Iwasaki et al., 2014; Wuehr et al., 2016; Chen et al., 2021; Fujimoto et al., 2021; Wuehr et al., 2024a; Fujimoto et al., 2018; Sprenger et al., 2020), 2 with stroke-induced hemiplegia (Horikawa et al., 2024; Bonan et al., 2016), one with MS (Lotfi et al., 2021), one with progressive supranuclear palsy (Wuehr et al., 2024b), one with UVP (Ceylan et al., 2021), and one with PPPD (Woll et al., 2019).

**Table 1:** Methodological characteristics and main results of these included studies.

References	Diagnosis	Sample size	Mean age (years), Gender (M/F)	GVS parameters	Postural assessments	Findings
Wuehr et al. (2022)	PD	*n* = 15	Mean age = 61.1 Gender = 11/4	*Location*: bilateral mastoid process *Galvanic current*: Zero-mean Gaussian white noise stimulation *Frequency*: 0–30 Hz *Intensity*: 0–0.7 mA	Posturographic force plate: sway velocity	nGVS-induced removals of body sway compatible with SR were discovered within 10 sufferers (67%) having optimal developments of 23% ± 13%
Pal et al. (2009)	PD	*n* = 5 (PD) *n* = 20 (healthy subjects)	PD (Mean age = 70, Gender = 3/2) healthy subjects (Mean age = 36.9, Gender =9/11)	*Location*: bilateral mastoid process *Galvanic current*: The present waveform was crafted utilizing a bespoke program developed within Matlab software *Frequency*: null *Intensity*: 0–0.5 mA	Force platform: RMS displacement in both the anteroposterior and mediolateral planes was meticulously recorded for each trial during COP measurement derivations.	Stochastic GVS applied at low intensities can effectively diminish sway in patients with PD
Samoudi et al. (2015)	PD	*n* = 10	Mean age = 61 Gender = 6/4	*Location*: bilateral mastoid process *Galvanic current*: Zero-mean white noise stimulation *Frequency*: 0–30 Hz *Intensity*: 0.5 mA	UPDRS-III, movement times, static posturography and force plate measurements of correcting response to a balanced perturbation	nGVS effectively enhanced balance adjustment following a rearward disturbance, and significantly reduced the latency of the postural response.
Peto et al. (2024)	PD	*n* = 32	Mean age = 68.1 Gender = 25/7	*Location*: bilateral mastoid process *Galvanic current*: Zero-mean Gaussian white noise stimulation *Frequency*: 0–30 Hz *Intensity*: 0–0.7 mA	Sway velocity, gait velocity, swing phase, stride time variability, stride time asymmetry, and base of support	Assessment of personal balance reactions revealed that 59% participants exhibited a favorable balance reaction following nGVS therapy, boasting an average optimal enhancement of 23%. Nevertheless, the ideal nGVS intervention did not impact gait parameters.
Kataoka et al. (2016)	PD	*n* = 5	Mean age = 68.6 Gender = 1/4	*Location*: bilateral mastoid process *Galvanic current*: Binaural monopolar stimulation *Frequency*: null *Intensity*: 0.7 mA	A pull test according to item 12 of the revised UPDRS part 3	Approximately 60% of patients diagnosed with Parkinson’s disease, who exhibited postural instability and/or abnormal axial posture, experienced an improvement in their postural stability subsequent to GVS application
Khoshnam et al. (2018)	PD	*n* = 11	Mean age = 67 Gender = 7/4	*Location*: bilateral mastoid process *Galvanic current*: electrical current stimulation *Frequency*: null *Intensity*: cutaneous sensory threshold	Step duration coefficient of variation, gait phase, phase coordination index, tapping score, and the number and duration of manual motor blocks	Nearly all assessed outcome measures saw enhancements upon the implementation of GVS, particularly with marked improvements in the fluctuation indices for step length consistency and tapping accuracy, and number of manual motor blocks
Okada et al. (2015)	PD	*n* = 7	Mean age = 68.6 Gender = 3/4	*Location*: bilateral mastoid process *Galvanic current*: Binaural monopolar stimulation *Frequency*: null *Intensity*: 0.7 mA	The mean anterior flexion angles, recorded in degrees, during a 30-s period with eyes both open and closed while maintaining a standing position	Following this GVS, there was a notable decrease in the anterior bending angles, both when the eyes were open and when they were closed.
Wuehr et al. (2023)	BVP	*n* = 11	Mean age = 54 Gender = 4/7	*Location*: bilateral mastoid process *Galvanic current*: Zero-mean Gaussian white noise stimulation *Frequency*: 0–30 Hz *Intensity*: 0–0.7 mA	Posturography: COP motion, the RMS of COP movement, and envelopment area traced by COP; Berg Balance Scale 6DOF motion platform: vestibular perceptual thresholds	In contrast to sham stimulation, the application of optimal nGVS diminished the body sway velocity by 25 ± 14%, decreased RMS of body sway by 22 ± 18%, and reduced the body sway area by 32 ± 26%. Notably, enhancements in vestibular perception due to nGVS were evident in 8 out of 11 patients (73%).
Eder et al. (2022)	BVP	*n* = 12 (nGVS + VRT) *n* = 11 (sham + VRT)	nGVS + VRT (Mean age = 61.92, Gender = 8/4) sham + VRT (Mean age = 62.64, Gender = 6/5)	*Location*: bilateral mastoid process *Galvanic current*: Zero-mean Gaussian white noise stimulation *Frequency*: 0–30 Hz *Intensity*: 0–0.7 mA	Posturography: postural stability, the mean velocity of sway; gait performance: walking velocity, base of support, and the coefficient of variation of stride time; functional gait assessment, the Timed Up and Go Test	Merging nGVS with VRT failed to elicit any impact on the evaluated outcome metrics, neither following 2 weeks of training nor at the subsequent 2-week follow-up evaluation
Schniepp et al. (2018)	BVP	*n* = 12	Mean age = 58.83 Gender = 6/6	*Location*: bilateral mastoid process *Galvanic current*: Zero-mean Gaussian white noise stimulation, sinusoidal stimulation *Frequency*: 0–30 Hz *Intensity*: 80% of the cutaneous threshold, 0–1.9 mA	Body motion responses, linear acceleration and angular speed of head and trunk segments	Supplementary nGVS contributed to the enhanced handling of subtle subthreshold vestibular inputs, consequently leading to a marked reduction in the vestibulospinal threshold in 90% of patients suffering from residual BVP
Iwasaki et al. (2018)	BVP	*n* = 12 (BVP) *n* = 19 (healthy subjects)	BVP (Mean age = 56.3, Gender = 9/3) healthy subjects (Mean age = 45.5, Gender =9/10)	*Location*: bilateral mastoid process *Galvanic current*: Zero-mean white noise stimulation *Frequency*: 0.02–10 Hz *Intensity*: 0–1 mA	The gait velocity, stride length, stride time, lateral movement distance, vertical movement distance, and the stride time’s coefficient of variation, lateral movement distance, vertical movement distance	The superior stimulus enhanced gait speed by 12.8% ± 1.3%, increased stride length by 8.3% ± 1.1%, and reduced stride duration by 3.7% ± 7% among individuals suffering from BVP
Iwasaki et al. (2014)	BVP	*n* = 11 (BVP) *n* = 21 (healthy subjects)	BVP (Mean age = 46.4, Gender = 6/5) healthy subjects (Mean age = 38.7, Gender =11/10)	*Location*: bilateral mastoid process *Galvanic current*: Zero-mean white noise stimulation *Frequency*: 0.02–10 Hz *Intensity*: 0–1 mA	Mean velocity of movement of COP, the envelopment area traced by a movement of COP, and RMS of COP distance.	nGVS enhanced the velocity, expanded the envelopment zone, and reduced RMS of COP in 91% of BVP patients, while also refining velocity, area, and RMS metrics. by 29.4% ± 4.9, 45.6% ± 4.7, and 22% ± 3.3%
Wuehr et al. (2016)	BVP	*n* = 13	Mean age = 50.1 Gender = 8/5	*Location*: bilateral mastoid process *Galvanic current*: Zero-mean Gaussian white noise stimulation *Frequency*: 0–30 Hz *Intensity*: 80% of cutaneous threshold	Stride time, stride length, base of support, double support time percentage and bilateral phase coordination index, and the coefficient of variation of stride time, stride length, and base of support	nGVS enhanced the stride time coefficient of variation by 26.0% ± 8.4%, optimized the stride length coefficient of variation by 26.0% ± 7.7%, and improved the base of support coefficient of variation by 27.8% ± 2.9%, as well as elevated the phase coordination index by 8.4% ± 8.8%
Chen et al. (2021)	BVP	*n* = 10 (BVP) *n* = 16 (healthy subjects)	BVP (Mean age = 51.3, Gender = 1/9) healthy subjects (Mean age = 40.9, Gender =8/8)	*Location*: bilateral mastoid process *Galvanic current*: Zero-mean the white noise stimulation *Frequency*: 0.02–10 Hz *Intensity*: 0–1 mA	Standing stability on a force plate: chest-pelvic ratio and lateral deviation of the center of mass	Under well-lit conditions, the lateral deviation of the center of mass was notably reduced during straight walking when subjects utilized nGVS for the BVP. In contrast, under dark conditions, the BVP subjects showed a diminished lateral deviation while employing nGVS.
Fujimoto et al. (2021)	BVP	*n* = 13	Mean age = 63.1 Gender = 8/5	*Location*: bilateral mastoid process *Galvanic current*: Zero-mean the white noise stimulation *Frequency*: 0.02–10 Hz *Intensity*: 0–1 mA	Posturography: mean velocity for COP movement, area enclosed by COP movement (area), and the RMS of COP displacement were calibrated within the XY plane	Subjective evaluations numbered 83 (64%) for unchanged and 33 (26%) for slightly improved
Wuehr et al. (2024a)	BVP	*n* = 19 (BVP) *n* = 15 (healthy subjects)	BVP (Mean age = 59.9, Gender = 10/9) healthy subjects (Mean age = 57.7, Gender =8/7)	*Location*: bilateral mastoid process *Galvanic current*: Zero-mean Gaussian white noise stimulation *Frequency*: 0–30 Hz *Intensity*: 0–0.7 mA	Posturographic force plate: velocity of body sway	nGVS-induced reductions of body sway compatible with SR were revealed in 12 sufferers (63%) with optimal improvements of 31% ± 21%. Within 10 participants (constituting 53%), the reductions in sway caused by nGVS surpassed the minimal threshold of clinical significance (with an optimal enhancement of 35% ± 21%), suggesting a pronounced SR effect.
Fujimoto et al. (2018)	BVP	*n* = 13	Mean age = 63.1 Gender = 8/5	*Location*: bilateral mastoid process *Galvanic current*: Zero-mean the white noise stimulation *Frequency*: 0.02–10 Hz *Intensity*: 0.1–1 mA	The mean velocity of the COP movement, the area enclosed by COP movement, and RMS of COP displacement	nGVS enhanced the COP’s velocity, coverage, and RMS metrics for a duration of 6 h subsequent to the termination of the stimulus
Ko et al. (2020)	BVP	*n* = 7 (BVP) *n* = 10 (healthy subjects)	BVP (Mean age = 53.4, Gender = 0/7) healthy subjects (Mean age = 29.1, Gender = 7/3)	*Location*: bilateral mastoid process *Galvanic current*: Zero-mean Gaussian white noise stimulation *Frequency*: null *Intensity*: 0–1 mA	AMTI force plate: RMS of COP sway, head rotation quality when walking with a 2 Hz head yaw	RMS of COP sway was considerably diminished in GVS while standing in BVP patients. While strolling, the 2 Hz head yaw motions were notably enhanced following the application of noGSV
Sprenger et al. (2020)	BVP	*n* = 30 (BVP) *n* = 24 (healthy subjects)	BVP (Mean age = 62.33, Gender = null) healthy subjects (Mean age = 61.29, Gender = null)	*Location*: bilateral mastoid process *Galvanic current*: White noise, low-and high-intensity perceptible stimulation *Frequency*: 0.02–20 Hz *Intensity*: 80% of the current at perception threshold, 0.5 mA, 1.5 mA	Force platform: displacement of COP in the medial-lateral and anterior–posterior directions, postural sway speed	No substantial disparities in postural sway velocity were observed between the nGVS and sham or noGVS groups. Furthermore, nGVS exhibited no significant stabilizing influence on posture in comparison to the noGVS or sham conditions.
Horikawa et al. (2024)	Stroke-induced hemiplegia	*n* = 22	Mean age = 66.36 Gender = 13/9	*Location*: bilateral mastoid process *Galvanic current*: Asymmetric pulse wave stimulation *Frequency*: 0–10 Hz *Intensity*: 3 mA	Assessment of righting reaction: COP measurement, Joint angle	During the corrective postural responses directed toward the paralyzed side, the application of right cathodal GVS augmented these righting movements. Conversely, when the postural righting response was toward the right side, the right cathodal GVS elicited a resistance to these corrective actions.
Bonan et al. (2016)	Stroke-induced hemiplegia	*n* = 35 (Stroke) *n* = 27 (healthy subjects)	Stroke (Mean age = 54.1, Gender = 22/13) healthy subjects (Mean age = 51.7, Gender =14/13)	*Location*: bilateral mastoid process *Galvanic current*: Trapezoidal stimulation *Frequency*: null *Intensity*: 2 mA	Force platform: lateral displacement of the COP, mean position of the COP in the mediolateral axis	GVS can adjust hemiparetic’s COP and their postural implications are linked
Lotfi et al. (2021)	MS	*n* = 24	Mean age = 41.87 Gender = 7/17	*Location*: bilateral mastoid process *Galvanic current*: Zero-mean Gaussian white noise stimulation *Frequency*: 0–30 Hz *Intensity*: 90% of the cutaneous sensory	The Sensory Organization Test: composite score in anteroposterior and lateral directions, Activities-Specific Balance Confidence Scale	The nGVS showed not improvements in composite score in anteroposterior and lateral directions and Activities-Specific Balance Confidence Scale total score
Wuehr et al. (2024b)	PSP	*n* = 16	Mean age = 70.7 Gender = 10/6	*Location*: bilateral mastoid process *Galvanic current*: Zero-mean Gaussian white noise stimulation *Frequency*: 0–30 Hz *Intensity*: 0–0.7 mA	Posturographic force plate: sway velocity, COP trajectory	The nGVS treatment significantly diminished body sway in line with SR, resulting in peak improvements of 31 ± 10% in 9 patients (constituting 56% of the group). Additionally, in 8 patients (50%), the reduction in sway due to nGVS surpassed the minimum threshold of clinical significance, showcasing an enhancement of 34% ± 5%
Woll et al. (2019)	PPPD	*n* = 24 (PPPD) *n* = 23 (healthy subjects)	PPPD (Mean age = 50.23, Gender = 8/16) healthy subjects (Mean age = 44.3, Gender = 10/13)	*Location*: bilateral mastoid process *Galvanic current*: White noise, low- and high-intensity perceptible stimulation *Frequency*: 0.02–20 Hz *Intensity*: 80% of the current at perception threshold, 0.5 mA, 1.5 mA	Posturography: The displacement of COP in medial–lateral and anterior–posterior directions were documented and the sum vector calculated, stabilogram diffusion analyses, postural sway speed	Postural sway speed decreased with nGVS compared to no current, low and high intensity current GVS.
Ceylan et al. (2021)	UVP	*n* = 41 (GVS + VRT) *n* = 32 (VRT)	GVS + VRT (Mean age = 36.05, Gender = 25/17) VRT (Mean age = 36.44, Gender = 19/16)	*Location*: bilateral mastoid process *Galvanic current*: Rectangular wave *Frequency*: 100 Hz *Intensity*: 1–5 mA	Equilibrium score, somatosensory sense score, center of gravity, visual sense score, vestibular sense score, Preferential sense	Equilibrium score, visual and vestibular sense scores, preference score, and center of gravity have a higher degree of improvement in the GVS group

BVP, bilateral vestibulopathy; GVS, galvanic vestibular stimulation; nGVS, noise galvanic vestibular stimulation; COP, center of pressure; VRT, vestibular rehabilitation training; UVP, unilateral vestibulopathy; PD, Parkinson’s Disease; PPPD, persistent postural-perceptual dizziness; PSP, progressive supranuclear palsy; RMS, root mean square; MS, multiple sclerosis.

#### PD

PD ranks as the second most prevalent neurodegenerative ailment, frequently accompanied by a decline in motor abilities, evidenced by symptoms including a gradually worsening asymmetric resting tremor, cogwheel-like rigidity, bradykinesia, and instability in posture (Simon et al., 2020). Postural instability ranks as one of the most incapacitating symptoms experienced by patients with PD, resulting in diminished mobility and frequent falls (Fasano et al., 2017). Falls are a crucial indicator for assessing life quality in sufferers with PD, and they represent one of the primary reasons for hospital admissions among this patient population (Martignoni et al., 2004). Although dopaminergic treatments can enhance postural stability in patients with PD, their efficacy is constrained, and prolonged usage may result in unwelcome adverse effects (Curtze et al., 2015; Sethi, 2008). Furthermore, procedures like deep brain stimulation, have been utilized to mitigate specific symptoms, albeit their invasive nature and variable efficacy, which is less efficacious or potentially detrimental for dynamic postural equilibrium (Szlufik et al., 2018; Wang et al., 2017). In the past few years, non-invasive approaches like GVS have been explored as potential alternative treatments. Seven articles applied GVS as a complementary therapy for postural control (Table 1; Wuehr et al., 2022; Pal et al., 2009; Samoudi et al., 2015; Peto et al., 2024; Kataoka et al., 2016; Khoshnam et al., 2018; Okada et al., 2015).

In this systematic review, a total of 85 patients with PD were encompassed. Each study employed electrode positioning above the bilateral mastoid processes. Nevertheless, it is worth noting that two studies utilized a pair of cathodes over the mastoids, while an anode was placed over the C7 vertebrae or the median aspect of both forearms (Pal et al., 2009; Khoshnam et al., 2018). Additionally, there were two studies that included stimulation of the trapezius muscles, in conjunction with the mastoid stimulation, as part of their protocol (Kataoka et al., 2016; Okada et al., 2015). For the characteristics of stimulation, three of the seven studies used galvanic current of zero-mean white noise stimulation (Wuehr et al., 2022; Samoudi et al., 2015; Peto et al., 2024) and two used binaural monopolar stimulation over the mastoids (Kataoka et al., 2016; Okada et al., 2015). White noise stimulation, spanning 0 to 30 Hz, was administered. Among the seven studies examined, four employed a current intensity that fluctuated between 0 and 0.7 mA (Wuehr et al., 2022; Peto et al., 2024; Kataoka et al., 2016; Okada et al., 2015), while the remaining two utilized a current intensity ranging from 0 to 0.5 mA (Pal et al., 2009; Samoudi et al., 2015). Solitary remaining investigation employed stimulation intensities that were tailored to each participant’s individual cutaneous sensory threshold (Khoshnam et al., 2018). Pertaining to the evaluations of postural control, seven papers delved into parameters pertinent to the sphere of balance, while two others explored parameters linked to the sphere of gait (Peto et al., 2024; Khoshnam et al., 2018). Regarding the impact form GVS on postural control, the unanimous consensus among all studies is that GVS yields advantageous outcomes for individuals with PD. The findings signified a notable enhancement in both postural control and balance.

#### BVP

BVP patients may experience either partial or total impairment of the peripheral vestibular system, resulting in persistent vertigo, notably an unsteady gait and equilibrium disturbances, particularly in dim lighting or on irregular terrain (Strupp et al., 2017), which significantly diminishes their health-linked quality of life metrics and heightens falls likelihood (Schniepp et al., 2017). Thus far, the scope of efficacious treatments for BVP remains narrow. At present, the sole therapeutic avenue capable of markedly enhancing the outcomes for those afflicted with BVP is vestibular rehabilitation therapy. This intervention is designed to enhance balance through the cultivation of multisensory postural control mechanisms, thereby compensating for and supplanting the diminished vestibular function (Hall et al., 2016). Nevertheless, the long-term outlook for BVP remains guarded, with therapeutic alternatives currently restricted to physical rehabilitation, which, at best, offers only incomplete restoration of the compromised vestibular function (Porciuncula et al., 2012). Recently, subtle white noise GVS (nGVS), imperceptible to the senses, has been employed to alter vestibular perception and enhance performance capabilities (Lajoie et al., 2021). Therefore, GVS could potentially emerge as a promising non-invasive therapeutic alternative for individuals suffering from peripheral vestibular hypofunction. Among all the included articles in this systematic review, there were 12 BVP studies that applied GVS as a non-invasive intervention (Table 1; Wuehr et al., 2023; Ko et al., 2020; Eder et al., 2022; Schniepp et al., 2018; Iwasaki et al., 2018; Iwasaki et al., 2014; Wuehr et al., 2016; Chen et al., 2021; Fujimoto et al., 2021; Wuehr et al., 2024a; Fujimoto et al., 2018; Sprenger et al., 2020).

A comprehensive systematic review encompassed 174 participants suffering from BVP. Each study incorporated electrode positioning above both mastoid processes, employing galvanic current with white noise stimulation. Five of the 12 studies used the frequency ranged from 0 to 30 Hz (Wuehr et al., 2023; Eder et al., 2022; Schniepp et al., 2018; Wuehr et al., 2016; Wuehr et al., 2024a), five used the frequency ranged from 0.02 to 10 Hz (Iwasaki et al., 2018; Iwasaki et al., 2014; Chen et al., 2021; Fujimoto et al., 2021; Fujimoto et al., 2018), and one used the frequency ranged from 0.02 to 20 Hz (Sprenger et al., 2020). Six of the 12 studies used present electrical intensity fluctuated within the range of 0 to 1 mA (Ko et al., 2020; Iwasaki et al., 2018; Iwasaki et al., 2014; Chen et al., 2021; Fujimoto et al., 2021; Fujimoto et al., 2018), three used the current intensity ranged from 0 to 0.7 mA (Wuehr et al., 2023; Eder et al., 2022; Wuehr et al., 2024a), and utilized three distinct stimulation intensities, each tailored to correspond with the individual’s cutaneous sensory threshold (Schniepp et al., 2018; Wuehr et al., 2016; Sprenger et al., 2020). Regarding the evaluations of postural control, a total of 11 studies examined parameters pertinent to the realm of balance (Wuehr et al., 2023; Eder et al., 2022; Schniepp et al., 2018; Iwasaki et al., 2014; Wuehr et al., 2016; Chen et al., 2021; Fujimoto et al., 2021; Wuehr et al., 2024a; Fujimoto et al., 2018; Sprenger et al., 2020), while an additional 2 studies focused on parameters associated with the sphere of gait (Eder et al., 2022; Iwasaki et al., 2018). For the effect of GVS on postural control, 10 of the 12 studies (83.3%) show that GVS have improved postural control (Wuehr et al., 2023; Schniepp et al., 2018; Iwasaki et al., 2018; Iwasaki et al., 2014; Wuehr et al., 2016; Chen et al., 2021; Fujimoto et al., 2021; Wuehr et al., 2024a; Fujimoto et al., 2018). However, two studies showed that GVS had not found any effect on the postural control (Eder et al., 2022; Sprenger et al., 2020).

#### Stroke-induced hemiplegia

Stroke-induced brain injury has the potential to compromise both postural and dynamic stability (Geiger et al., 2001). Enhancing balance in the aftermath of a stroke is imperative, as it is inextricably linked to the elevation of patient independence and the enhancement of their overall quality of life. Vestibular caloric stimulation can improve postural bias in patients with hemiparetic (Rode et al., 1998). Thus, vestibular stimulation has emerged as an effective intervention strategy for enhancing postural stability in individuals affected by hemiplegia subsequent to a stroke. In this systematic review, there are 2 studies reporting the use of GVS to affect posture of stroke-induced hemiplegia patient (Table 1; Horikawa et al., 2024; Bonan et al., 2016).

In this systematic review, a cohort of 57 individuals suffering from hemiplegia secondary to stroke was examined. Electrodes were meticulously secured to the bilateral mastoid processes of each participant. For stimulation characteristics, Bonan et al. (2016) used galvanic current of trapezoidal stimulation and the current intensity 2 mA. Horikawa et al. (2024) used galvanic current of asymmetric pulse wave stimulation, the frequency ranged from 0 to 10 Hz, and the current intensity 3 mA. In relation to the postural control assessments, these investigations revealed that GVS has the capacity to regulate the COP and enhance righting responses in individuals suffering from post-stroke hemiplegia.

#### Other neurological diseases

A few other studies have also explored use of GVS in MS, PSP, PPPD, and UVP. Thus, a total of 24 patients with MS, 16 PSP, 24 PPPD, and 73 UVP were encompassed within these present systematic reviews (Table 1; Ceylan et al., 2021; Lotfi et al., 2021; Wuehr et al., 2024b; Woll et al., 2019).

All investigations necessitate the positioning of electrodes above the bilateral mastoid processes. For the characteristics of stimulation, three studies used galvanic current of white noise stimulation (Lotfi et al., 2021; Wuehr et al., 2024b; Woll et al., 2019) and a study employed rectangular wave stimulation on the mastoid processes for its investigation (Ceylan et al., 2021). To highlight the properties of stimulation, two investigations employed galvanic current in the form of white noise stimulation, which encompassed a frequency spectrum from 0 to 30 Hz, as documented in Lotfi et al. (2021) and Wuehr et al. (2024b). Frequency of rectangular wave stimulation is 100 Hz (Ceylan et al., 2021). Two investigations employed stimulation intensities determined by each participant’s unique cutaneous sensory threshold (Lotfi et al., 2021; Woll et al., 2019). The PSP study employed a current intensity spectrum from 0 to 0.7 mA, whereas the UVP investigation operated within a current intensity bracket of 1 to 5 mA (Ceylan et al., 2021; Wuehr et al., 2024b). Three studies show that GVS significantly improved postural control and balance of PSP, PPPD, and UVP (Ceylan et al., 2021; Wuehr et al., 2024b; Woll et al., 2019). However, Lotfi et al. (2021) not found any effect on the postural control and balance of MS.

### Risk of bias assessment

The methodological excellence, as evaluated by NOS scores, spans from a rating of 2 to 5. Based on the aggregate points awarded, 20 studies (accounting for 80%) were deemed to have a minimal risk of bias (Wuehr et al., 2023; Ceylan et al., 2021; Wuehr et al., 2022; Pal et al., 2009; Peto et al., 2024; Kataoka et al., 2016; Ko et al., 2020; Eder et al., 2022; Schniepp et al., 2018; Iwasaki et al., 2018; Iwasaki et al., 2014; Wuehr et al., 2016; Chen et al., 2021; Fujimoto et al., 2021; Wuehr et al., 2024a; Sprenger et al., 2020; Horikawa et al., 2024; Bonan et al., 2016; Wuehr et al., 2024b; Woll et al., 2019), whereas 5 investigations (constituting 20%) exhibited a significant risk of bias (Samoudi et al., 2015; Khoshnam et al., 2018; Okada et al., 2015; Fujimoto et al., 2018; Lotfi et al., 2021; Supplementary Table 1).

## Discussion

This comprehensive review examined the impact of GVS on the postural stability and equilibrium of individuals with neurological conditions. Our analysis revealed that GVS generally exerted a positive influence on postural equilibrium across numerous studies. Moreover, it is noteworthy that the studies incorporated within this review exhibited considerable variability in the GVS protocols employed, supplementary interventions, and the demographics of the populations studied, potentially influencing the drawn inferences.

In this systematic review, GVS protocols used between studies were inconsistent. According to galvanic current type, GVS can be divided into noisy GVS (nGVS) and non-nGVS. The noisy GVS effects operate on the principle of stochastic resonance (SR), which is at the core of their mechanism. SR refers to the occurrence wherein a noisy input, possessing a mean value distinct from zero and operating below the threshold of human perception, harnesses the power of chaotic numerical sequences to refine the sensory nervous system, thereby enhancing the absorption and integration of external information into the organism (Collins et al., 1995). The application of SR has been extensively implemented across diverse sensory receptors within the human body, significantly enhancing the regulation of lower limb posture (Reeves et al., 2009). In this systematic review, we found that nGVS and non-nGVS significantly contributed to adjust the balance responses of PD patients in all studies. Nevertheless, the study conducted by Peto et al. (2024) revealed that the optimal nGVS failed to elicit any impact on gait parameters. The researcher posits that the effects induced by nGVS along the ascending pedunculopontine nucleus-thalamo-cortical pathways are congruent with a notable therapeutic response for postural symptoms, whereas they yield a negligible or non-existent response in the gait deficits associated with Parkinson’s disease. This suggests that nGVS primarily aids in the regulation of static balance rather than enhancing locomotion (Mahmud et al., 2022). On the contrary, Further research demonstrated that the coefficient of variation for step duration was notably enhanced following the administration of GVS in individuals with PD (Khoshnam et al., 2018). The refined gait enhancement noted in the research could likely be ascribed to GVS exerting its influence on the striatum via the vestibular nerve’s afferent pathways. Besides, these divergent results may be related to differences in GVS protocols. Peto et al. applied nGVS. Khoshnam et al. chosen the current stimulus to be a direct current. It is recognized that the responses to GVS treatment are contingent upon the specific stimulation parameters employed, which can vary from one patient to another. Additional studies are essential to comprehensively delineate the impacts of GVS and ascertain its long-term effectiveness in the management of PD.

Thus far, the options for treating BVP have been rather restricted. In the past few years, efforts have been directed toward enhancing and stimulating the remaining vestibular excitability in BVP patients through the use of unperceived vestibular noise stimulation via non-invasive nGVS (Wuehr et al., 2017). In the current systematic review, we reviewed the impacts of GVS stimulation on the postural stability in individuals with BVP. White noise stimulation was used in all included studies. We found that nGVS effectively improves postural control in most studies. There are several possible mechanisms that could explain the results. As mentioned before, It was postulated that the enhancements in both static and dynamic balance observed in BVP following nGVS intervention might be attributable to SR mechanism (Moss et al., 2004). SR serves as the underlying mechanism by which nonlinear systems amplify their response to a faint signal amidst noise (Collins et al., 1995). Given that the vestibular system inherently operates in a nonlinear fashion (Sadeghi et al., 2007), the introduction of noisy GVS may augment the activation of the vestibulospinal pathways via SR. This enhancement might subsequently amplify the stimulatory signals directed toward the antigravity muscles of the lower extremities, dependent upon the phase, thereby improving postural control (Bent et al., 2004). Nevertheless, Eder and colleagues conducted a placebo-controlled, double-blind clinical trial, wherein they integrated nGVS with conventional standardized vestibular rehabilitation training (VRT) to investigate the potential for enhanced therapeutic synergy between the two interventions. Although VRT typically led to moderate enhancements in the balance abilities of patients, the study revealed no indication that augmenting VRT with nGVS conferred any supplementary benefits to their balance proficiency (Eder et al., 2022). The lack of synergistic interaction between nGVS and VRT could be associated with the mechanism of imperceptible stochastic vestibular stimulation. Studies have demonstrated that nGVS is particularly effective in enhancing the detection of subtle, subthreshold vestibular signals and in bolstering vestibular-related balance capabilities during stationary stance or gradual head movements (Eder et al., 2022). Consequently, nGVS did not impact the vestibular-related perception of suprathreshold stimuli nor did it influence balance functionality when encountering dynamic equilibrium challenges. Additionally, the lack of observed effects of nGVS on balance performance and confidence may be attributed to the specific timing of the assessment following the treatment. Eder and colleagues conducted their initial evaluation of the therapeutic outcomes over 12 h subsequent to the final administration of nGVS, potentially overlooking any nGVS-induced impacts that may have arisen during the stimulation process or in the immediate aftermath. Upon comparing nGVS with appropriate control conditions (noGVS, sham), Sprenger et al. (2020) failed to verify the advantageous impact of nGVS on postural stability in BVP. They think that a adequate control stimulation conditions was a prerequisite for achieving reliable results from GVS study. Therefore, further research needs to be done to confirm these findings.

GVS adeptly adjusts vestibular cortical regions by harnessing afferent inputs to stimulate the vestibular system (Lopez et al., 2012). An accumulating body of scholarly work is delving into the utilization of GVS as a method to rectify postural instability in stroke survivors. The study conducted by Horikawa et al. (2024) revealed that GVS has the capacity to alter the center of sitting pressure and the joint angles involved in postural correction reactions. The sitting balance function is closely linked with stroke recovery outcomes and the capacity to carry out daily activities; GVS could potentially be utilized as an instrument to enhance sitting balance effectively, in a manner that is both safe and straightforward. In like manner, a separate study utilizing repetitive GVS as a therapeutic approach for addressing left or right hemispheric lesions has shown the potential to adjust COP in hemiparetic individuals, with the postural consequences being significantly correlated (Bonan et al., 2016). These studies suggest that GVS may be a potential therapeutic treatment for stroke-induced hemiplegia. Besides, some studies have explored the effect of postural control in MS, PSP, PPPD, and UVP in this systematic review. Initial postural instability and mysterious recurring falls are pivotal to the clinical manifestation of PSP (Höglinger et al., 2017). Imbalance issues in PSP typically stem from a complex interplay of factors, potentially encompassing trunk stiffness, ocular motility impairments, and dysfunction in the vestibular balance reflexes (Liao et al., 2008). A recent investigation revealed that the application of nGVS for vestibular neuromodulation achieved significant clinical improvements in reducing postural instability for nearly half of the evaluated patients suffering from PSP (Wuehr et al., 2024b). Similar results have been found in PPPD and UVP (Ceylan et al., 2021; Woll et al., 2019). However, the opposite results were found in the MS (Lotfi et al., 2021). One main reason for these findings might be the small sample size that made it difficult to detect significant changes. Another reason was that there is no optimal interval between the nGVS sessions. Further research is needed to expand sample and improve design to assess the efficacy of nGVS in enhancing balance among patients with MS.

### Strengths and limitations

The review’s merits are evident in its employment of a comprehensive and methodical search approach, which incorporates a diverse array of search terms as keywords to broaden the review’s coverage. Additionally, the search strategy was devoid of any limitations regarding the research’s temporal or geographical boundaries. In addition, most studies stated a clear purpose and had a poor risk of bias (NOS scores ≥ 3). All scholarly inquiries reported detailed clinical information.

This assessment highlights certain constraints. Due to the variability in clinical aspects and methodologies among the studies under review, a quantitative synthesis of the outcomes (meta-analysis) was not feasible. Additionally, the limited sample size presents a constraint in studies assessing the application of GVS for enhancing body balance control. Moreover, our research did not encompass studies documented in languages other than English, potentially limiting the available evidence on this subject.

## Conclusion

GVS emerges as a promising adjunctive treatment, significantly enhancing postural stability and equilibrium in adult patients suffering from neurological conditions. Despite several decades of investigation into GVS as a method for enhancing balance, the body of high-quality research in this area remains limited. Future research needs to provide more consideration of the homogeneity of samples recruited, comparative control group, adopt *a priori* sample size calculations, select targeted outcome measures, and parameters of the GVS to improve research quality.

## Data Availability

The original contributions presented in the study are included in the article/Supplementary material, further inquiries can be directed to the corresponding author.
