# Supplementary material for: Galvanic vestibular stimulation for postural rehabilitation in neurological disorders: a systematic review
Source: Front Neurosci. 2025 Apr 16;19:1580078. doi: 10.3389/fnins.2025.1580078 (PMC12040823; doi:10.3389/fnins.2025.1580078)
Supplement: Supplementary file 1 [file Table_1.docx]

**Supplementary Table S1 Methodological quality assessment**

|  | **Authors, year** | **Sample representativeness** | **Intervention protocol** | **Comparability** | **Outcome** | **Statistics** | **Score** |
| --- | --- | --- | --- | --- | --- | --- | --- |
| **PD** | Wuehr et al., 2022 | **√** | **√** |  | **√** | **√** | **4** |
|  | Pal et al., 2009 |  | **√** | **√** | **√** |  | **3** |
|  | Samoudi et al., 2015 |  | **√** |  | **√** |  | **2** |
|  | Peto et al., 2024 | **√** | **√** |  | **√** | **√** | **4** |
|  | Kataoka et al., 2016 |  | **√** |  | **√** | **√** | **3** |
|  | Khoshnam et al., 2018 |  | **√** |  | **√** |  | **2** |
|  | Okada et al., 2015 |  | **√** |  | **√** |  | **2** |
| **BVP** | Wuehr et al., 2023 |  | **√** | **√** | **√** | **√** | **4** |
|  | Eder et al., 2022 | **√** | **√** | **√** | **√** |  | **4** |
|  | Schniepp et al., 2018 |  | **√** |  | **√** | **√** | **3** |
|  | Iwasaki et al., 2018 | **√** | **√** | **√** | **√** |  | **4** |
|  | Iwasaki et al., 2014 |  | **√** | **√** | **√** | **√** | **4** |
|  | Wuehr et al., 2016 |  | **√** |  | **√** | **√** | **3** |
|  | Chen et al., 2021 |  | **√** | **√** | **√** |  | **3** |
|  | Fujimoto et al., 2021 |  | **√** |  | **√** | **√** | **3** |
|  | Wuehr et al., 2024 | **√** | **√** | **√** | **√** | **√** | **5** |
|  | Fujimoto et al., 2018 |  | **√** |  | **√** |  | **2** |
|  | Ko et al., 2020 |  | **√** | **√** | **√** |  | **3** |
|  | Sprenger et al., 2020 | **√** | **√** | **√** | **√** |  | **4** |
| **stroke-**  **induced hemiplegia** | Bonan et al., 2016 | **√** | **√** | **√** | **√** |  | **4** |
|  | Horikawa et al., 2024 |  | **√** | **√** | **√** |  | **3** |
| **MS** | Lotfi et al., 2021 |  | **√** |  | **√** |  | **2** |
| **PSP** | Wuehr et al., 2024 |  | **√** |  | **√** | **√** | **3** |
| **PPPD** | Woll et al., 2019 | **√** | **√** | **√** | **√** |  | **4** |
| **UVP** | Ceylan et al., 2021 | **√** | **√** | **√** | **√** |  | **4** |
